# Increased Body Fat and Organic Acid Anions Production Are Associated with Larger Kidney Size in ADPKD

**DOI:** 10.3390/medicina58020152

**Published:** 2022-01-19

**Authors:** Adriana dos Santos Dutra, Fernanda Guedes Rodrigues, Daniel Ribeiro da Rocha, Larissa Collis Vendramini, Ana Cristina Carvalho de Matos, Ita Pfeferman Heilberg

**Affiliations:** 1 Nutrition Post Graduation Program, Universidade Federal de São Paulo, São Paulo 04023-062, Brazil; adsd.dutra@gmail.com (A.d.S.D.); fernanda.gr91@gmail.com (F.G.R.); 2 Nephrology Division, Universidade Federal de São Paulo, São Paulo 04023-062, Brazil; danielribroc@gmail.com (D.R.d.R.); larissavendramini.uscs@yahoo.com (L.C.V.); anacrismatos21@gmail.com (A.C.C.d.M.)

**Keywords:** ADPKD, body fat, organic acid anion, total kidney volume, adiposity markers, obesity, renal function decline

## Abstract

*Background and Objectives*: A high body mass index (BMI) is associated with the progression of autosomal dominant polycystic kidney disease (ADPKD). However, body fat (BF), which is another adiposity marker, has not yet been studied. Excessive weight may promote elevation in the endogenous synthesis of organic acid (OA) anions. Accordingly, we aimed to investigate the possible association of the aforementioned markers with kidney volume and renal function in patients with ADPKD. *Materials and Methods*: We conducted a retrospective cohort study of adult ADPKD outpatients involving clinical, serum, and urinary laboratorial data and body composition assessments retrieved from their medical records. BF was estimated by skinfold thickness (mm) on the non-dominant arm and was considered as normal or high for each sex. Total kidney volume (TKV) and height-adjusted volume (htTKV) were measured by magnetic resonance imaging. The annual estimated glomerular filtration rate (eGFR) slope was analyzed during a median follow-up time of 6 (5.0–7.0) years to calculate rapid progression (decline in renal function ≥2.5 mL/min/year over 5 years). *Results*: A total of 104 patients were included (41.9 ± 11.9 years old, 38.5% men), with 62.5% of the patients classified as high BF. The High BF group presented higher levels of OA, glycosylated hemoglobin (HbA1c), C-reactive protein (CRP), 24 h urinary sodium (UNa), and htTKV, and lower eGFR than those with a normal BF. In the multivariate linear regression, the associated variables with TKV were high BF, OA and BMI (std. β 0.47, *p* < 0.05; std. β 0.36, *p* = 0.001; std. β 0.25, *p* = 0.01, respectively). In the binary logistic regression, when adjusted for potential confounders, UNa was the only parameter associated with an increased risk of eGFR decline ≥2.5 mL/min/year (OR 1.02, 95% CI 1.01–1.03, *p* = 0.02). *Conclusions*: Increased body fat and endogenous production of organic acid anions are associated with larger kidney size in ADPKD but not with a decline in renal function.

## 1. Introduction 

Autosomal dominant polycystic kidney disease (ADPKD) is the most common genetic nephropathy, characterized by innumerable progressively growing kidney cysts that destroy the renal tissue structure, leading to progressive loss of renal function [1]. The prevalence is estimated to be at least 9 cases per 10,000 individuals [2]. ADPKD is considered the fourth leading cause of end-stage renal disease (ESRD), which occurs in half of the patients with ADPKD during or after the sixth decade of life and is responsible for the need for renal replacement therapy in 5% to 10% of patients [3,4].

Despite its genetic origin, several factors, including clinical, epigenetic, laboratorial, radiological, and environmental factors, are considered predictors of disease progression among patients with ADPKD. Male gender, gross hematuria, nephrolithiasis, type of mutation, proteinuria, and inflammation may worsen the progression of the disease [5,6]. Among the etiopathogenic environmental features affecting the severity of ADPKD, high salt and protein intake, caffeine consumption, smoking, level of fluid intake, and higher body mass index (BMI) stand out [7,8,9,10,11,12,13]. The interaction among all these prognostic factors is related to the increase in the number and size of cysts, resulting in an increase in the total kidney volume, which is the most important surrogate marker of progression [4].

The association between BMI and the progression of ADPKD was recently pointed out by Nowak et al. [12] whose *post hoc* analysis of the Halt Progression of Polycystic Kidney Disease (HALT) study showed that overweight and, particularly, obesity were associated with increased kidney volume and loss of renal function in patients with early-stage ADPKD. Subsequently, the same investigators, using data from another cohort (TEMPO 3:4 trial), reported that a higher BMI was associated with a greater annual increase in kidney volume, but not with renal function decline [13].

Metabolic syndrome, insulin resistance, kidney hyperfiltration, albuminuria, increased vasopressin, and derangements in glucose and lipids metabolism activating pathways that increase cyst growth may all contribute to the connection between obesity and a worse ADPKD prognosis [14]. However, other underlying mechanisms remain to be established. Excessive body weight may be implicated in an increased endogenous production of organic acids [15,16].

Traditionally, BMI is considered a surrogate marker of adiposity; notwithstanding, it cannot discriminate between fat mass and fat-free mass [17,18,19,20]. Accordingly, we hypothesized that body composition, particularly body fat, may play an important role in the progression of ADPKD. We aimed to investigate the possible association between adiposity markers, such as body fat and endogenous organic acids production, with kidney volume and renal function in ADPKD patients.

## 2. Materials and Methods

This is a retrospective observational study, based on medical records of ADPKD outpatients at the Polycystic Kidney Disease Outpatient Clinic of the Universidade Federal de São Paulo (UNIFESP), between 2012 and 2018. Adult patients (>18 years old) with estimated glomerular filtration rate (eGFR) > 15 mL/min/1.73 m^2^, with available anthropometric and body composition parameters and biochemistry data, were selected for inclusion. The diagnosis of ADPKD was confirmed by renal ultrasonography, according to the criteria of Pei et al. [21]. Exclusion criteria were: pregnancy and severe heart failure. The study protocol was approved by the Ethics Advisory Committee at UNIFESP (3588.704/2019). 

### 2.1. Anthropometry and Body Composition

Weight (kg) and height (m) were measured to calculate body mass index (BMI), defined as body weight in kilograms by height squared in meters (kg/m^2^). To avoid overestimating the contribution of kidney size to BMI, body weight was adjusted by subtracting kidney weight, estimated by magnetic resonance (MRI) (see below), assuming a tissue density equal to that of water (1 g/cm^3^) [22]. 

The percentage of body fat (%BF) was estimated by skinfold thickness (mm) using the equation of Durnin and Womersley [23]. Skinfold measurements were performed at four sites (biceps, triceps, subscapular, and suprailiac) on the non-dominant arm using the Lange skinfold caliper (Cambridge Scientific Instruments, Cambridge, MD, USA). Three sets of measurements were averaged for each site. High body fat was considered ≥25% for men and ≥32% for women, according to Lohman et al. [24].

### 2.2. Nutritional Data

The body surface area (BSA)-dependent organic acid (OA) anion component, reflecting primarily endogenous acids production, was calculated by the formula of Berkemeyer and Remer [16]: OA (mEq/d) = body surface area (m^2^) × 41/1.73; body surface area (BSA) (m^2^) = 0.007184 × height (cm) 0.725 × weight (kg) 0.425. Sodium chloride (NaCl) daily intake was estimated from 24 h urinary sodium excretion (UNa) according to the formula: NaCl (g/day) = UNa (mEq/24 h)/17 [25]. Protein intake was estimated from urinary urea through the protein equivalent of nitrogen appearance (PNA) formula: PNA = 9.35 × G (mg/min) + 11.04 and urea nitrogen generation (G) = urinary urea (mg/L) × urinary volume per 24 h (L)/2.14/1440 [26].

### 2.3. Clinical and Biochemical Parameters 

Hypertension at admission was defined based on office or clinic levels of systolic pressure ≥ 140 mmHg and diastolic pressure ≥ 90 mmHg [27] or the use of antihypertensive drugs. Metabolic syndrome was defined according to the National Cholesterol Education Program Adult Treatment Panel III Guidelines (NCEP-ATP III) [28], withdrawing the criterion of waist circumference to avoid bias by the increased size of the kidneys. According to the NCEP-ATP III, cutoff values for normal high density lipoprotein cholesterol levels were ≥40 mg/dL (men) and ≥50 mg/dL (women) and <150 mg/dL for triglycerides.

Biochemical parameters considered were serum creatinine, fasting glucose, glycated hemoglobin (HbA1c), low-density lipoprotein (LDL) cholesterol, high-density lipoprotein cholesterol (HDL), triglycerides, C-reactive protein (CRP), and 24 h urinary urea, Na, and osmolality. Creatinine serum was determined using a modified Jaffe reaction with a calibration traceable to reference by mass spectrometry with isotopic dilution (ID-MS) [29].

### 2.4. Imaging and Renal Function

Baseline total kidney volume (TKV) was measured by magnetic resonance imaging (MRI) with renal volumetrics performed by obtaining length, width, and depth using the ellipsoid equation. Values were combined from both kidneys, corrected for height (htTKV) [30]. Age and htTKV were used to identify the risk of progression in the Mayo imaging subclasses, as slow (1A and 1B) or rapid risk of progression (1C, 1D, 1E) [30].

The Chronic Kidney Disease Epidemiology Collaboration equation (CKD-EPI) [31] was used to estimate glomerular filtration rate (eGFR). Decline in renal function was assessed through the calculation of the annual slope of eGFR (mL/min/1.73 m^2^) over 5 years. Our analysis was restricted to patients exhibiting three to seven measurements of eGFR from baseline determinations. Rapid progression was considered when the eGFR slope was greater than or equal to 2.5 mL/min/year over 5 years [32].

### 2.5. Statistical Analysis

Statistical analysis was performed using IBM SPSS version 23.0 (SPSS Inc., Chicago, IL, USA). In all analyses, *p* < 0.05 was considered significant. The variables’ distribution was evaluated by the Kolmogorov–Smirnov test. Categorical variables are presented as n (%), normally distributed variables as mean ± standard deviation (SD), and non-normally distributed variables as median (interquartile range (IQR)). Residuals were checked for normality, and variables were natural log-transformed when appropriate. Patients were divided into two groups according to normal or high body fat percentage, and differences between them were tested by Student’s *t*-test or Mann-Whitney, as appropriate. Pearson’s chi-squared or Fisher’s exact tests were applied between categorical variables. The slope was calculated individually based on the least squares method, which consists of an estimator that minimizes the sum of squares of the linear regression residuals. Linear regression analysis was performed to study possible determinants of TKV and binary logistic regression to assess potential factors associated with an eGFR decline ≥ 2.5 mL/min/year. All variables with a *p*-value < 0.20 in the univariate analysis were subsequently included in the multivariate model, adjusted for age, sex, presence of hypertension, and baseline eGFR.

## 3. Results

A total of 235 medical records of ADPKD patients were reviewed, of whom 59 had incomplete parameters of body composition data, 48 did not have all available biochemistry data, and 24 had an eGFR ≤ 15 mL/min/1.73 m^2^. A total of 104 subjects met the inclusion criteria (64 women and 40 men, 41.9 ± 11.9 years old). Clinical and laboratorial parameters of all patients were obtained. Hypertension was significantly higher among high BF patients (81.3 vs. 64.1%). As shown in Table 1, 65 (62.5%) of the patients presented with high BF (mean BF of 36.7% ± 6.2%), with no statistical difference regarding sex (*p* = 0.09). The high BF group presented with higher levels of OA, HbA1c, and CRP, and lower eGFR than the normal BF group, with no significant differences between groups regarding diabetes, metabolic syndrome, mean values of fasting glucose, blood lipids, and urine osmolality. The percentage of patients with abnormal serum levels of glucose, HDL, and triglyceride also did not differ. Patients in the high BF group consumed more NaCl (11.5 ± 3.6 g/day vs. 9.5 ± 4.2 g/day, *p* = 0.02) than those in the normal BF group. Protein intake assessed by PNA did not differ between the groups. Of 104 patients, 76 had available data for TKV calculation. 

**Figure 1:**
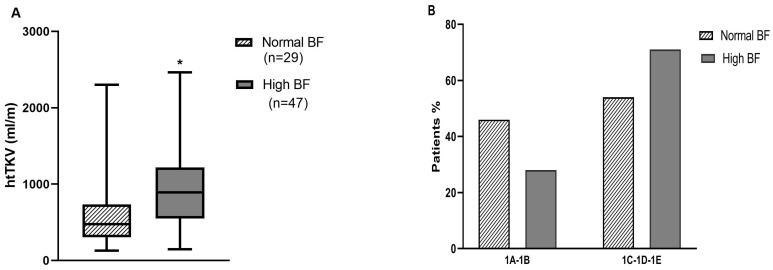
(**A**) htTKV according to body fat (normal BF, high BF) according to htTKV (Mann-Whitney test applied, * *p* = 0.01). (**B**) Mayo clinic risk classes (1A-1B or 1C-1D-1E) according to body fat (Pearson’s chi-squared test applied).

As illustrated in Figure 1A, htTKV was statistically higher among patients with high BF (914.8 mL/m, interquartile range (IQR): 607.4, 1242.5 vs. 412.6 mL/m, interquartile range (IQR): 302.8, 745.0, *p* = 0.01). According to the Mayo imaging classification, 63.2% were rapid progressors (1C-1D-1E) but there was no significant difference with respect to body fat percentage between these slow and rapid progressors (Figure 1B). 

In order to study possible determinants of TKV, we performed a linear regression analysis, as shown in Table 2. The univariate analysis revealed significant and direct associations between TKV and high BF, OA, and BMI. When adjusted for potential confounders such as age, sex, presence of hypertension, and eGFR, these variables remained independently associated with TKV, as evidenced in the multivariate analysis.

Of 76 patients with MRI imaging evaluated, 52 had a median follow-up of 6 years (IQR: 5.0, 7.0) (data not shown in tables). The results of binary logistic regression using rapid progression (eGFR decline ≥ 2.5 mL/min/year over 5 years) as the dependent variable are shown in Table 3. After adjustment for potential confounders, urinary sodium (UNa) was the only parameter independently associated with rapid progression of ADPKD.

## 4. Discussion

Increased body mass has been recently shown to be independently associated with the rate of TKV growth in other cohorts [12,13]. Body weight and BMI represent well-defined and widely spread parameters of body adiposity [19,20,33,34] but measurements of percent body fat are preferable [18,35]. To the best of our knowledge, this is the first study to assess the role of body fat in total kidney volume and renal function decline among ADPKD patients. In the current series, 62.5% of ADPKD patients (39.4% overweight and 24.0% obese) were found to exhibit high body fat, which was directly associated with higher hTKV and TKV, but not with the decline in renal function.

The link between excessive body fat and increased kidney volume is not well-established yet, but some hypotheses have been raised. Obese individuals with metabolic syndrome present high plasma levels of copeptin [36], a marker of arginine-vasopressin (AVP), which is independently associated with ADPKD progression [37]. However, the association of BMI with kidney growth in ADPKD is not associated with plasma copeptin levels [13]. Obesity may activate mTOR complex 1 and inhibit AMP-activated kinase (AMPK), resulting in cystic growth [38,39]. Experimental data showed that conditional cilia mutant mice become obese and hyperphagic, and develop cystic kidney disease [40]. In addition, cystic cells reprogram their metabolism to use aerobic glycolysis to provide energy [41] so that caloric restriction ameliorates the course of the disease [42]. Metabolic defects in glucose metabolism, mitochondrial abnormalities, and impaired utilization of fatty acids are seen in ADPKD [41,43]. van Gastel and Meijer [14] recently questioned whether the contribution of higher BMI to the progression of ADPKD is related to the increase in body size *per se* or to the concomitant metabolic syndrome. In the current study, the prevalence of metabolic syndrome was low (around 17%) and did not differ between patients with high or normal body fat. It is worth mentioning that waist circumference was not included in the metabolic syndrome criteria to avoid bias by increased kidney size. We did not observe statistical differences between the groups regarding the percentage of patients with high triglyceride or low HDL levels, the latter being a known parameter of progression [8]. The percentage of patients under statin treatment was not high and also did not differ between the groups. Nevertheless, patients in the high BF group had higher levels of HbA1c, pointing to a possible early alteration in glucose homeostasis and insulin resistance due to the increase in body fat, regardless of the low rate of diabetes mellitus diagnosis.

Obesity may cause inflammation [44] and it may worsen the course of ADPKD, including at the early stages of the disease [45]. We noticed a significantly higher mean value of CRP in the high BF group, and an association with TKV was also disclosed in the univariate but not in the multivariate analysis.

Unmeasured anion accumulation and retention and increased acid synthesis are hallmarks of obesity, and higher BMI has been associated with an increased risk of developing anion gap metabolic acidosis [15,46]. Here, we noticed a strong and direct association between the endogenous production of organic acid anions (OA), BMI, and body fat, and TKV, despite adjusting for potential confounders such as age, sex, presence of hypertension, and baseline measured eGFR, in the multivariate analysis. A previous cross-sectional analysis of the baseline data from the Halt Progression of Polycystic Kidney Disease (HALT) study conducted by Torres et al. [47] showed that body surface area (BSA) was independently associated with baseline height-adjusted TKV. However, given that OA was estimated by anthropometrics (calculated based on BSA) in the current series, we only considered TKV (and not hTKV). In the present series, the group with a high BF consisted of 55 out of 65 (84.7%) subjects with a BMI ≥ 25 kg/m^2^. Furthermore, metabolic acidosis is a common complication of moderate to severe CKD and has been shown to increase the risk of CKD progression; as eGFR decreases, there is less excretion of ammonia and alkalis [48,49]. Interestingly, Blijdorp et al. [50] reported an inverse association between serum bicarbonate and risk of eGFR decline or renal failure in a cohort of ADPKD patients. Organic acid anions reduce urinary pH, and an inverse relationship between urinary pH and body weight has been reported in other populations [51,52], eventually due to insulin resistance [51]. Among ADPKD patients, reduced urinary pH has already been described [6,53,54], although the mechanisms are not fully elucidated, Torres et al. [55] proposed a urinary concentration defect as being responsible for decreasing ammonia transfer to urine, hence reducing urinary pH. Unfortunately, urinary pH data were not available in the present series. Furthermore, Blijdorp et al. [50] recently showed that ADPKD patients in the lowest tertile of serum bicarbonate had higher BMI and lower urine ammonium excretion, suggesting a possible interaction between acid excretion and higher body weight in this population. Metabolic acidosis is a trigger for urinary citrate excretion reduction [56], and hypocitraturia is frequently observed in ADPKD patients [6,53,54], suggesting urinary citrate as a possible prognostic factor in this disease [57]. Therefore, obesity is a clinical condition that is related to acid retention and, in this sense, it may contribute to a poorer prognosis in ADPKD.

A direct and independent association between urinary sodium (a marker of high NaCl intake) and a decline in eGFR ≥ 2.5 mL/min/year over 5 years, suggesting progression was evident in the present analysis. These findings are in line with other studies carried out in ADPKD patients, in which increased sodium excretion was associated with a decline in renal function [7,58]. The possible explanation is that the increase in NaCl intake leads to ADPKD progression by vasopressin stimulation [7,37].

In the current series, there was no association between body adiposity, represented by high BF, organic acids, and BMI with the eGFR decline during the analyzed period of over 5 years. Although observed in a small sample, our results are similar to findings reported by Nowak et al. [13] in a much larger sample.

Limitations of the present study include the retrospective observational nature of the study, not being able to establish a cause–effect relationship, and the possible selection bias in the sampling. The availability of data as just one TKV measure, performed at the beginning of the study, without the possibility of studying the influence of this measure over time, is also a limiting factor. The absence of urinary pH, citrate, and serum bicarbonate data precluded further analysis of the acid-base status and its relationship with obesity and adiposity. In addition, the level of physical activity could not be assessed. Finally, the relatively small number of patients can also be viewed as a limiting factor in our analysis. Our study also has strengths. Although body composition data were not obtained by a gold standard method such as DEXA [35], skinfold thickness measurements are considered a valid method of estimating the percentage of body fat, providing a simple, low-cost, and less invasive method to determine the fat reserve [18]. In addition, its correlation with gold-standard methods is strong and was validated in CKD patients [35]. The inference of body adiposity must be used with caution, not considering only BMI, as it does not properly differentiate body composition. Given its clinical relevance, body fat should be incorporated into prognostic models to predict renal outcomes in patients with ADPKD and explored as a treatment target, since it represents a modifiable risk factor. 

Current data encourage future studies with a larger sample and longer follow-up to validate our findings, in addition to the inclusion of other body composition methods such as DEXA or computed tomography, which allow for the evaluation of the effect of muscle mass on these outcomes.

## 5. Conclusions

The present study suggests that increased body fat and endogenous production of organic acid anions are associated with greater kidney size in ADPKD, irrespective of the presence of fully established metabolic syndrome, but they are not independent risk factors for the decline in renal function.

## Figures and Tables

**Table 1:** Clinical and laboratory parameters of ADPKD patients according to body fat percentage.

		% Body Fat (BF)		
Variable	Total n = 104	Normal BF n = 39	High BF n = 65	*p*
Age, years	41.1 ± 11.9	35.6 ± 11.3	44.4 ± 11.1	<0.001
Sex, Female/Male, n (%)	64 (61.5)/40 (38.5)	20 (51.3)/19 (48.7)	44 (67.7)/21 (32.3)	0.09
Body Fat, %	32.2 ± 8.1	25.1 ± 5.1	36.7 ± 6.2	<0.001
BMI (kg/m^2^)	26.7 ± 4.8	23.3 ± 3.2	28.6 ± 4.2	<0.001
OA, mEq/day	43.4 ± 4.8	41.3 ± 5.2	44.6 ± 4.2	0.01
Diabetes, n (%)	5 (4.7)	1 (2.6)	4 (6.2)	0.65
Metabolic syndrome, n (%)	16 (17.2)	5 (15.2)	11 (18.3)	0.70
Statins use, n (%)	20 (19.2)	5 (12.8)	15 (23.1)	0.20
Hypertension, n (%)	79 (76.0)	25 (64.1)	54 (83.1)	0.03
Laboratorial parameters				
eGFR, mL/min/1.73^2^	77.6 (42.8–108.3)	89.0 (64.0–119.0)	67.0 (36.4–99.0)	0.01
Fasting glucose, mg/dL	93.4 ± 14.0	94.3 ± 18.7	93.3 ± 11.4	0.80
High glucose levels, n (%)	8 (7.7)	3 (11.5)	5 (9.6)	0.53
HbA1c, %	5.7 ± 0.7	5.2 ± 0.5	5.9 ± 0.8	0.01
Cholesterol LDL, mg/dL	111.7 ± 31.3	103.6 ± 34.2	113.9 ± 28.7	0.16
Cholesterol HDL, mg/dL	49.2 ± 13.9	51.3 ± 15.2	48.0 ± 13.3	0.30
Low HDL levels n (%)	41 (39.4)	9 (34.6)	32 (54.2)	0.09
Triglycerides, mg/dL	126.8 ± 61.7	114.9 ± 59.2	132.4 ± 62.7	0.22
High Triglyceride levels n (%)	22 (21.2)	7 (25.0)	15 (26.8)	0.86
CRP, mg/dL	0.23 (0.09–0.58)	0.13 (0.04–0.37)	0.31 (0.13–0.87)	0.01
UNa, mEq/day	182.6 ± 66.5	162.8 ± 70.8	195.4 ± 60.8	0.02
Urinary osmolality, mOsm/kg H_2_O	415.5 ± 149.9	407.6 ± 164.2	421.3 ± 139.3	0.70
Nutritional data				
PNA, g/day	70.2 ± 18.7	68.9 ± 18.0	71.2 ± 19.4	0.59
NaCl, g/day	10.8 ± 4.0	9.5 ± 4.2	11.5 ± 3.6	0.02

Variables are presented as mean ± standard deviation, number (% of total) or median (interquartile range) or as percentage for categorical variables. For continuous variables, Student’s *t*-test or Mann-Whitney was used, as appropriate. Pearson’s chi-squared test was applied for categorical variables normal BF: men < 25%, women < 32%; high BF: men ≥ 25%, women ≥ 32%; high glucose: ≥110 mg/dL; low HDL levels: men < 40 mg/dL, women < 50 mg/dL; high triglycerides: ≥150 mg/dL. Abbreviations: BMI, body mass index; OA, organic acid; eGFR, estimated glomerular filtration rate; HbA1c, hemoglobin A1c; LDL, low-density lipoprotein; HDL, high-density lipoprotein; CPR, C-reactive protein; PNA, protein equivalent of nitrogen appearance; UNa; urine sodium; NaCl: sodium chloride.

**Table 2:** Univariate and multivariate linear regression analysis using log TKV as the dependent variable.

	Univariate Analysis		Multivariate Analysis *	
Variables	Std. β	*p*	Std. β	*p*
High BF %, yes	0.34	0.03	0.47	<0.05
OA, mEq/day	0.56	<0.001	0.36	0.001
BMI, kg/m^2^	0.37	<0.01	0.25	0.01
CRP, mg/dL	0.19	0.10	0.17	0.11
PNA, g/day	0.13	0.29	-	-
UNa, mEq/day	0.09	0.45	-	-

n = 76; * Adjusted for age, sex, hypertension, and eGFR. Abbreviations: Std. β, standardized beta; BF, body fat; OA, organic acid; BMI, body mass index; CPR, C-reactive protein; PNA, protein equivalent of nitrogen appearance; UNa; urine sodium.

**Table 3:** Binary logistic regression analysis using annual eGFR slope decline ≥2.5 mL/min/year over 5 years as dependent variable.

	Univariate Analysis			Multivariate Analysis *		
	OR	95% IC	*p*	OR	95% IC	*p*
**Variables**						
OA, mEq/day	1.12	0.99–1.27	0.06	1.07	0.91–1.26	0.43
BMI, kg/m^2^	1.10	0.98–1.23	0.12	1.09	0.95–1.24	0.23
High BF %, yes	0.78	0.46–1.31	0.34	-	-	-
CRP, mg/dL	0.63	0.16–2.45	0.51	-	-	-
PNA, g/day	1.04	0.99–1.07	0.06	1.03	0.98–1.07	0.28
UNa, mEq/day	1.01	1.00–1.02	0.04	1.02	1.01–1.03	0.02
htTKV, mL/m	1.00	0.99–1.00	0.26	-	-	-

n = 52; * Adjusted for age, sex, hypertension and eGFR. Abbreviations: OR, odds ratio; CI, confidence interval; OA, organic acid; BMI, body mass index; BF, body fat; CPR, C-reactive protein; PNA, protein equivalent of nitrogen appearance; UNa; urine sodium; htTKV, height-adjusted total kidney volume.
